# Pharmacological PPARα Activation Markedly Alters Plasma Turnover of the Amino Acids Glycine, Serine and Arginine in the Rat

**DOI:** 10.1371/journal.pone.0113328

**Published:** 2014-12-08

**Authors:** Anette Ericsson, Nigel Turner, Göran I. Hansson, Kristina Wallenius, Nicholas D. Oakes

**Affiliations:** 1 Department of Bioscience, AstraZeneca R&D Mölndal, Mölndal, Sweden; 2 Garvan Institute of Medical Research, Sydney, New South Wales, Australia; 3 Department of Pharmacology, University of New South Wales, Sydney, Australia; Clermont Université, France

## Abstract

The current study extends previously reported PPARα agonist WY 14,643 (30 µmol/kg/day for 4 weeks) effects on circulating amino acid concentrations in rats fed a 48% saturated fat diet. Steady-state tracer experiments were used to examine *in vivo* kinetic mechanisms underlying altered plasma serine, glycine and arginine levels. Urinary urea and creatinine excretion were measured to assess whole-body amino acid catabolism. WY 14,643 treated animals demonstrated reduced efficiency to convert food consumed to body weight gain while liver weight was increased compared to controls. WY 14,643 raised total amino acid concentration (38%), largely explained by glycine, serine and threonine increases. ^3^H-glycine, ^14^C-serine and ^14^C-arginine tracer studies revealed elevated rates of appearance (*R_a_*) for glycine (45.5±5.8 versus 17.4±2.7 µmol/kg/min) and serine (21.0±1.4 versus 12.0±1.0) in WY 14,643 versus control. Arginine was substantially decreased (−62%) in plasma with estimated *R_a_* reduced from 3.1±0.3 to 1.2±0.2 µmol/kg/min in control versus WY 14,643. Nitrogen excretion over 24 hours was unaltered. Hepatic arginase activity was substantially decreased by WY 14,643 treatment. In conclusion, PPARα agonism potently alters metabolism of several specific amino acids in the rat. The changes in circulating levels of serine, glycine and arginine reflected altered fluxes into the plasma rather than changes in clearance or catabolism. This suggests that PPARα has an important role in modulating serine, glycine and arginine *de novo* synthesis.

## Introduction

Previous studies on PPARα agonists have focused on lipid and glucose metabolism and therefore knowledge of effects on amino acid metabolism is limited. One study in mice reported that the synthetic PPARα ligand WY 14,643 down-regulates several genes involved in amino acid metabolism in a PPARα dependent fashion [1]. We previously showed in a rat model of dyslipidemia and insulin resistance that WY 14,643 treatment had substantial effects on plasma amino acid levels and hepatic gene expression, the latter suggestive of a decreased amino acid catabolism and an increased polyamine synthesis [2]. Conservation of amino acids for local synthetic processes and production of polyamines, are compatible with events supporting ongoing growth and cell division occurring during liver enlargement, a well known effect induced by WY 14,643 in rodent [3].

Our overall aim was to further elucidate the effects of PPARα activation by WY 14,643 on whole body amino acid catabolism in fat-fed rats by addressing several specific questions. Are the marked WY 14,643 changes in plasma levels of glycine, serine and arginine due to alterations in rates of appearance? How does treatment affect whole amino acid catabolism and liver arginine metabolism?

To answer these questions, in vivo tracer studies, indirect calorimetry and nitrogen excretion measurements were performed.

## Materials and Methods

### Ethics statement

Experimental procedures were approved by the Local Ethics Review Committee on Animal Experiments (Göteborg Region, Sweden).

### Animals and treatment

Male Sprague Dawley rats (350–400 g, Harlan, Netherlands) had free access to food and water and were maintained on a 12-h light/dark cycle at 21–22°C. All animals were initially fed a carbohydrate-rich standard rodent chow diet (R3, Lantmännen, Stockholm, Sweden) for ∼1 week consisting of 26% protein, 12% fat, 62% carbohydrates by calories, with energy content ∼12.6 kJ/g. Thereafter animals were switched to a high fat diet containing 48% saturated fat, 16% casein of which protein content was specified as 95%. The diet also contained free methionine 0.3% (wt/wt) and had a total energy content of 21.4 kJ/g. Food intake and body weights were recorded daily beginning two days prior to start of fat feeding.

Following one week on fat diet rats received daily dosing with WY 14,643 (30 µmol/kg/day, Medicinal Chemistry, AstraZeneca R&D Mölndal, Sweden) or an equivalent volume of vehicle (2.5 ml/kg, 0.5% hydroxypropylmethylcellulose) by oral gavage at 13:00 each day.

### Blood samples and analyses

Blood samples were collected from the tail vein after 3 hours of fasting on three different occasions: after one week on chow diet; after one week on fat diet and; after 4 weeks of treatment (vehicle/WY 14,643) corresponding to 5 weeks on fat diet. Glucose, insulin, triglycerides (TG), free fatty acids (FFA) were were carried out as previously described [4]. Following the last blood sample the animals were used either for studies of amino acid turnover or for assessment of whole body fuel and energy metabolism (described below).

### Preparation of tracers and animals

Tracer infusate for serine/glycine turnover studies, for each rat ∼0.8×10^8^ dpm L-[U-^14^C]serine (^14^C-serine, Amersham Pharmacia Biotech, Uppsala, Sweden) and ∼2.0×10^8^ dpm [2-^3^H]glycine (^3^H -glycine, Amersham) were reconstituted together in 1.7 ml sterile normal saline. For arginine turnover studies, for each rat ∼0.65×10^8^ dpm L-[U-^14^C]arginine monohydrochloride (^14^C-arginine, Amersham) was reconstituted in 1.3 ml saline.

Animals (n = 6/group) were fasted for ∼9 h and then anaesthetized intraperitoneally with Na-thiobutabarbitol (Inactin®, RBI/Sigma, St. Louis, MO; 120 mg/kg) and were catheterized according to methods previously described [5]. Two basal blood samples were collected ∼10 min apart for analysis of plasma amino acid profile. Analysis of 22 amino acids were carried out as previously described [2].

### Tracer infusion protocol

^3^H-glycine and ^14^C-serine together or ^14^C-arginine (Amersham Pharmacia Biotech, Uppsala, Sweden) alone were given as a priming dose (120 µl/min for 1 min) followed by a constant infusion (17 µl/min). In glycine/serine turnover experiments arterial blood samples were collected 10, 30, 40, 60, 70 and 80 min after the start of tracer infusion. In arginine turnover studies samples were collected 20, 40 and 60 min after start of tracer. Following top-up dosing with anesthetic, dissection and weighing of extensor digitorum longus (EDL) muscles and liver were performed.

### Estimation of whole body turnover and clearance rates

Assuming metabolic stability and attainment of tracer steady state, the plasma clearance rate (*K*, in units of ml/min) and rate of appearance (*R_a_*, µmol/min) of the amino acids were calculated using standard non-compartmental equations [6]. For cases where steady state was not achieved during the tracer infusion period an estimate of the plateau tracer concentration was made based the assumption that the impulse response of the plasma compartment was described by a double exponential decay.

### Indirect calorimetry

Indirect calorimetry was performed in the 9–11 h fasting state (corresponding to the conditions/timing of the tracer studies) using a custom made system. Animals (n = 16/group) were acclimatized to the 5 l flow-through chambers for two hours the day prior to and one hour immediately before start of measurements. A constant flow of compressed dry air (1.5 l/min/cage) was provided by a mass-flow regulator (Bronkhorst, Germany) to a time multiplexed system via computer controlled 3-way valves with four test chambers and one empty reference chamber. Outflow gas was passed through a Permapure, Nafion dryer, (50 tubes 48″ SS shell, Omnifit, Cambridge, UK) using N_2_ as the drying gas. Measurements of O_2_ (oxygen analyzer S-3A/II, with R2 flow control and N-37M sensor, AEI Technologies Inc., Pittsberg, USA), CO_2_ (carbon dioxide analyzer SIFOR 200 range 0–2%, Maiak AG, Hamberg, Germany) and gas flow rates were made.

Based on the measured variables, individual mean values for oxygen consumption (*VO_2_*), carbon dioxide production (*VCO_2_*), and respiratory quotient (*RQ*) were calculated. Following completion of the measurements animals were returned to their home cages with 4-7 days elapsing before assessment of 24 h urinary nitrogen excretion (n = 8/group) or for collecting liver tissue for arginase activity measurements (n = 8/group), described below.

### Estimation of whole body energy expenditure and energy production from protein, carbohydrate and lipid oxidation in the fasted state

Whole body energy expenditure was calculated using the classic equation of Weir [7] based on *VO_2_* and *VCO_2_*. Mass substrate oxidation rates for carbohydrate, lipid and protein were calculated from *VO_2_* and *VCO_2_* as well as the *N* data, assuming constant elimination over 24 h, using equations from Even and Nadkarni [8] and then these estimates were converted using the caloric equivalents (in W) for individual fuels from Ferrannini [9].

### Urinary nitrogen excretion

Animals were housed in metabolic cages for 24 hours, with ad libitum access to food and water and all urine was collected and volumes (*V_u_*) measured. Concentrations of urinary urea (*C_urea_*, Horiba ABX, France) and creatinine (*C_creat_*, DiaSys Diagnostic Systems GmbH, Germany), were analysed and total nitrogen excretion (*N*) was calculated as *N = V_u_*(2x*C_urea_*+3x*C_creat_*) in units of mmol/day.

### Arginase activity in liver

Animals (n = 8/group) were euthanized with isoflurane during the 5^th^ week of treatment and livers collected, weights recorded and the tissue snap frozen in liquid nitrogen and stored at −80°C. Arginase activity was measured in liver homogenates using the thiosemicarbazide-diacetylmonoxime-urea assay based on a previously described method [10].

### Data presentation and statistics

All data are presented as means ± SEM where n equals the number of animals used in the analysis. The differences between vehicle and WY 14,643 treated animals were evaluated statistically by Student's t-test for unpaired observations. Paired Student's t-test was used to assess within animal dietary effects. A P-value <0.05 was considered significant.

## Results

### Body weight and food intake

**Figure 1:**
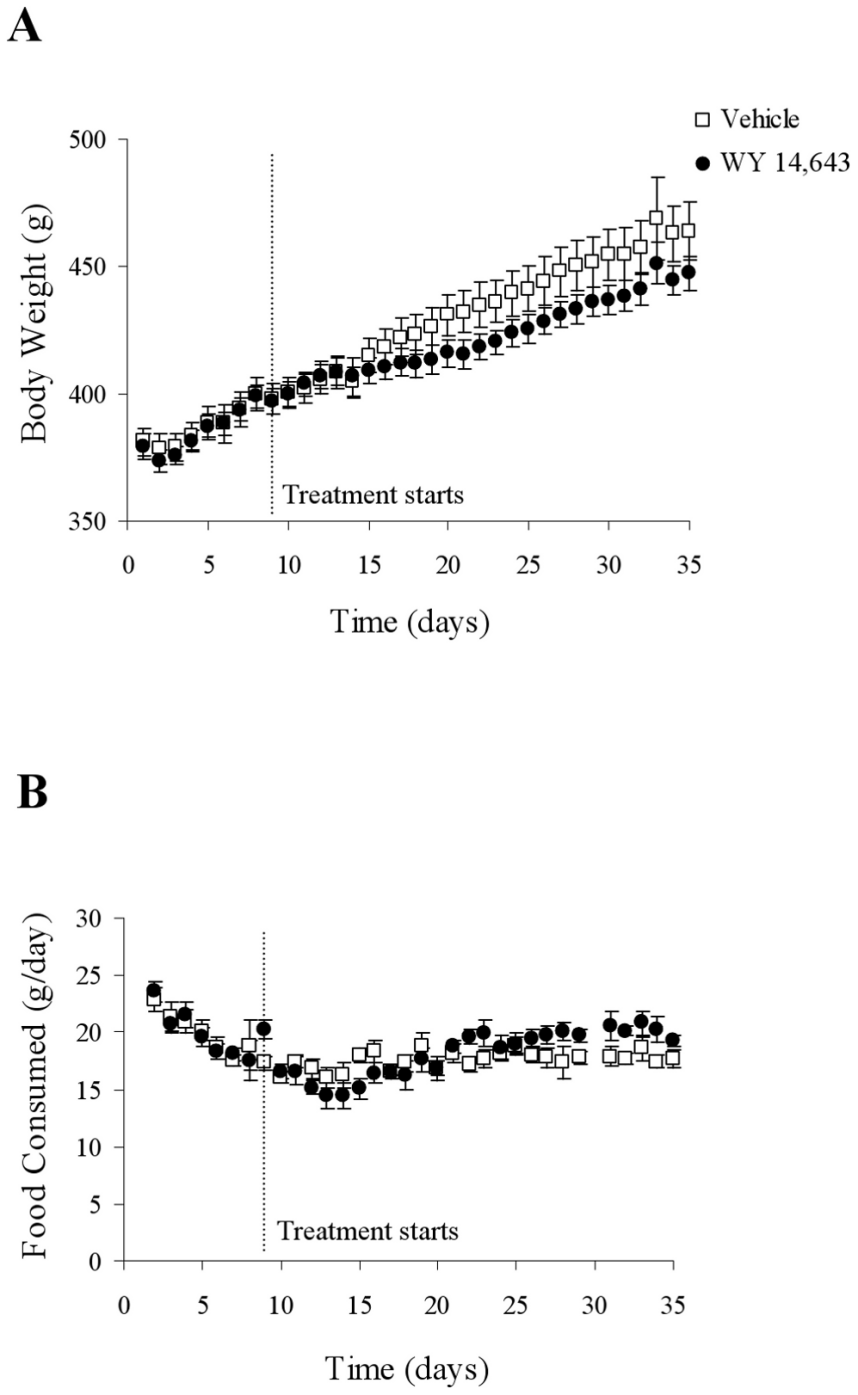
Body weight development (A) and consumption of high-fat diet (B). Rats were treated per orally with WY 14,643 (n = 10) or vehicle (n = 10). Values are given as means ± SEM.

Daily body weight and food intake data from the start of high-fat feeding are presented in Fig. 1. At treatment start, on day 9, the groups were matched for both food intake and body weight. WY 14,643 treated animals underwent a period of reduced body weight gain during the 2^nd^ week of treatment (Fig. 1A) which was then followed by a restoration of body weight gain (but no catch up) compared to the vehicle group. Over the whole treatment period body weight gain was on the borderline to being statistically lower in the WY 14,643 group (63.6±3.0 g, n = 10) versus control animals (82.8±8.7 g, n = 10, P = 0.05). Cumulative food intake was not significantly different in the WY 14,643 (488±14 g) versus vehicle (476±12 g) groups (Fig. 1B).

### Pharmacodynamic effects of PPARα agonism

Fat feeding resulted in persistent elevations in plasma insulin, TG and FFA levels and the HOMA insulin resistance index versus initial chow fed levels, seen after 1 week (Table 1). Following 4 weeks of WY 14,643 treatment plasma levels of insulin, TG, FFA decreased compared to controls and also a decrease in insulin resistance was observed. Plasma glucose, which is not elevated in response to high fat feeding, was slightly but significantly increased in the WY 14,643 compared to the vehicle control animals.

**Table 1:** Clinical chemistry data.

	Chow diet >1 wk		Fat diet 1 wk		Fat diet 5 wk, treatment 4 wk	
Plasma level	Vehicle	WY 14,643	Vehicle	WY 14,643	Vehicle	WY 14,643
Glucose (mM)	6.70±0.09	6.95±0.14	6.97±0.12	7.00±0.13	6.99±0.10	7.57±0.15*
Insulin (nM)	0.23±0.02	0.22±0.01	0.42±0.02#	0.42±0.02	0.42±0.02	0.32±0.02*
TG (mM)	0.67±0.03	0.64±0.05	1.84±0.12#	1.93±0.11	2.05±0.15	0.82±0.04*
FFA (mM)	0.48±0.03	0.41±0.02	0.63±0.03#	0.64±0.04	0.65±0.04	0.56±0.03*
Rel HOMA_IR_	1.00±0.07	1.02±0.07	1.92±0.11#	1.91±0.12	1.92±0.11	1.59±0.19*

Plasma factors in 3 hour fasted rats. TG, triglycerides; FFA, free fatty acids; Rel HOMA_IR_ (relative to Vehicle Chow diet group HOMA_IR_), insulin resistance index. Values are given as means ± SEM with n = 37 per group. #P<0.05 vehicle on fat diet for 1 week versus vehicle on chow diet. *P<0.05 WY 14,643 versus vehicle after 4 weeks of treatment.

Liver enlargement is a known effect of PPARα agonism in rodents and in the current study WY 14,643 for 4–5 weeks increased liver weight by 79%: from 14.2±0.3 g in vehicle controls to 25.5±0.8 g in rats treated with WY 14,643, P<0.05, n = 13. Another known effect at high doses of PPARα agonists is muscle wasting. However, EDL muscle mass was not different between groups (WY, 156±5 mg, n = 6; vehicle 165±6 mg, n = 6).

### Altered plasma amino acid levels in response to WY 14,643

**Figure 2:**
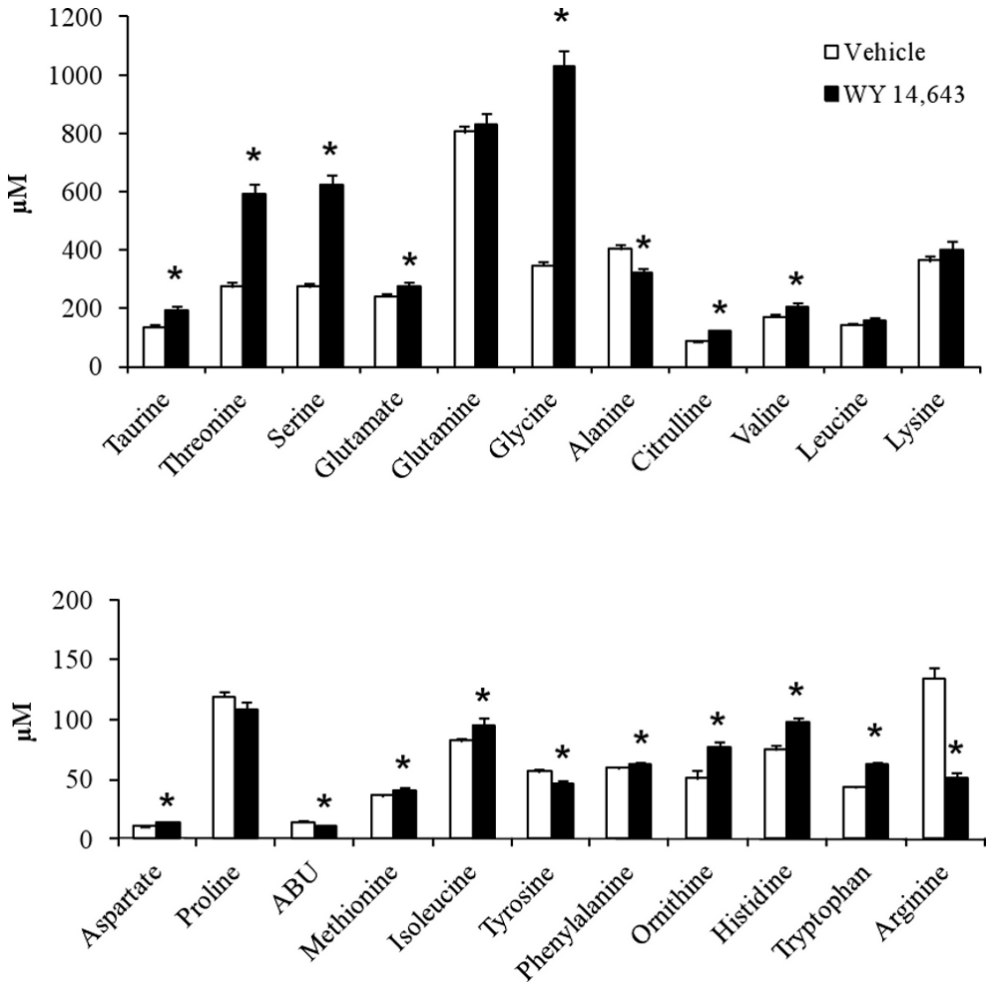
Plasma amino acid concentrations. Plasma samples were collected after 4 weeks of treatment with WY 14,643 (n = 15) or vehicle (n = 15) in 9 hour fasted rats. ABU, 2-aminobutyric acid. Values are given as means ± SEM. * P<0.05.

WY 14,643 induced increases in glycine and serine, as well as a marked reduction in arginine levels (Fig. 2) which was consistent with the results of our previous study [2]. HPLC analysis demonstrated that animals treated with WY 14,643 exhibited elevated taurine, aspartate, threonine, glutamate, citrulline, valine, isoleucine, ornithine, histidine, lysine and tryptophane. Other than arginine, only alanine, ABU (2-aminobutyric acid) and tyrosine were found to be decreased (Fig. 2). Total plasma concentrations of amino acids were 38% higher in WY 14,643 treated animals (5418±229 µM) as compared to control group (3916±123 µM, P<0.05).

### Serine, glycine and arginine clearance and rates of appearance in plasma

**Figure 3:**
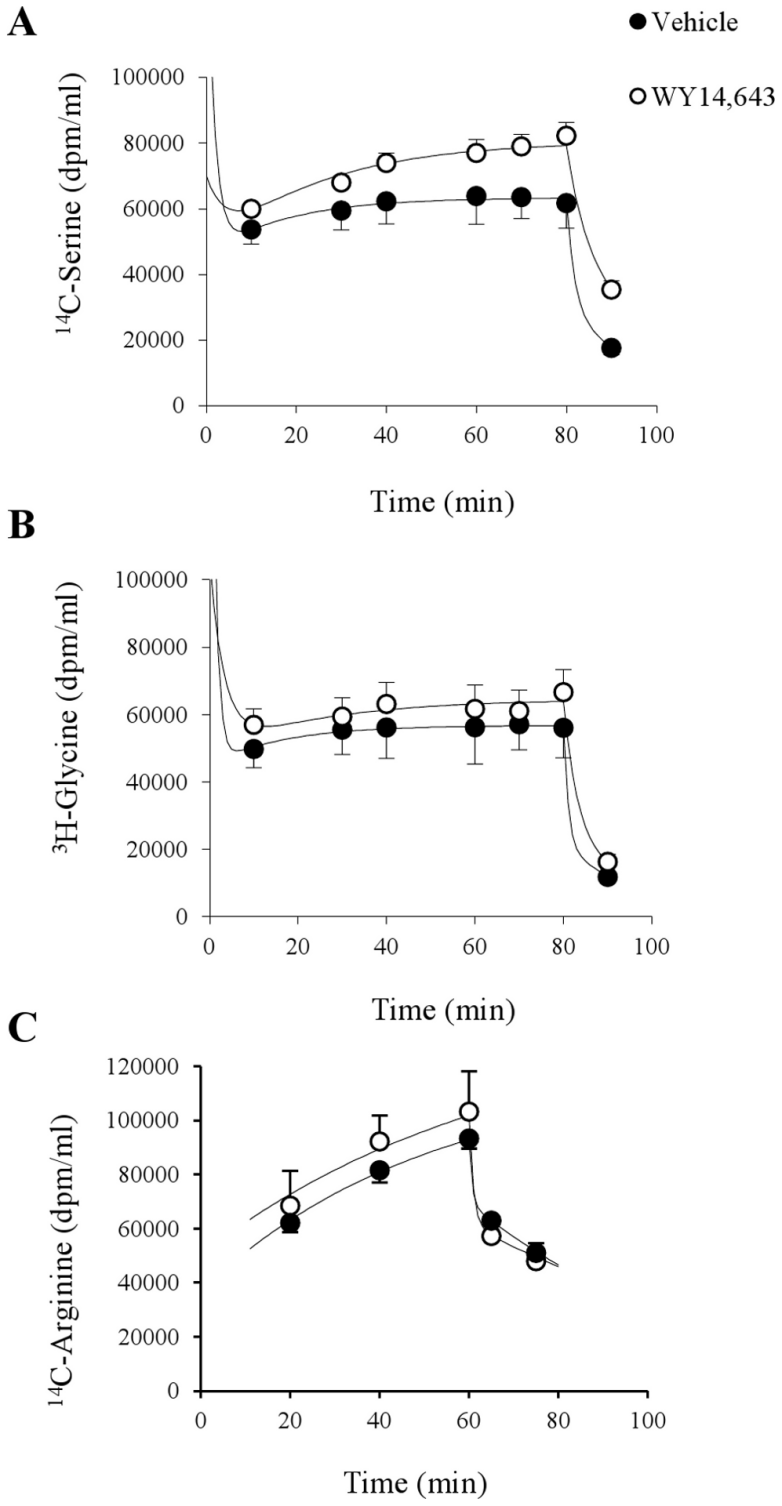
Normalised plasma concentrations of ^14^C-serine (A), ^3^H-glycine (B) and ^14^C-arginine (C) during intravenous infusion. Isotope labeled amino acids were given as a bolus (n = 6/group) to obtain steady state levels and kept at a constant infusion rate for 80 (A and B) and 60 (C) min duration, respectively. Values are given as means ± SEM.

**Figure 4:**
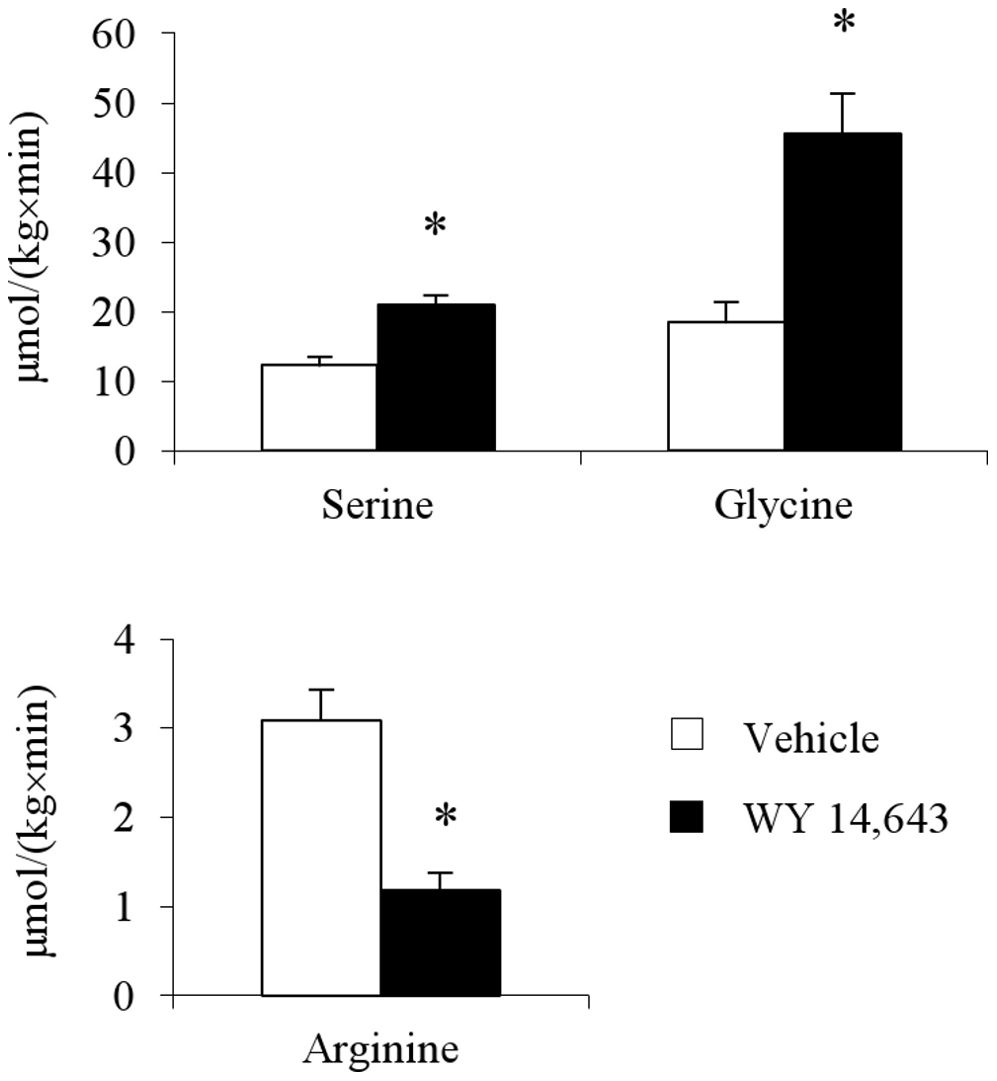
Rates of appearance (*R_a_*) of serine, glycine and arginine in plasma. Animals per group were n = 6. Values are given as means ± SEM. *P<0.05 versus vehicle.

Fig. 3 summarizes the plasma tracer levels in response to primed constant intravenous infusions of the three amino acid tracers. Results were normalized to a constant infusion rate of 1.0×10^6^ dpm/min levels (and the corresponding priming dose of ∼7×10^6^ dpm). Plasma ^14^C-serine approached a higher plateau level in the WY 14,643 compared to controls (Fig. 3A) reflecting a moderate treatment induced lowering of serine clearance: WY 14, 643, 31.9±1.1 versus Vehicle, 40.4±3.3 ml/kg/min, P<0.05. Estimated glycine clearance rates were similar in both groups: WY 14, 643, 43.0±6.4 versus Vehicle, 50.5±8.5 ml/kg/min. Calculated rates of appearance (*R_a_*) of the amino acids are summarized in Fig. 4 and reveal the major mechanism of the treatment increases in serine and glycine; increased rate of entry into plasma. Thus in the WY 14,643 group *R_a_* for serine was 1.7-fold the vehicle level which could explain the bulk of the 2.2-fold increase in plasma serine level with a smaller contribution from reduced plasma clearance. Similarly, in the WY 14,643 group *R_a_* for glycine was 2.5-fold the vehicle level comparable with the 2.9-fold increase in plasma glycine level.

By contrast plasma ^14^C-arginine data (Fig. 3C) failed to achieve steady state probably due to an insufficient priming dose. The plasma ^14^C-arginine levels at the 60 min time point had reached ∼75% of the extrapolated plateau value for the vehicle and WY14, 643 groups respectively. The whole body clearance and *R_a_* for arginine are probably in error (overestimated) by ∼30% with the likely impact on the difference between groups being much smaller. Bearing in mind the limitations, the clearance rate estimates based on the 60 min data were similar for vehicle 22.5±2.1 and WY 14,643 24.7±2.0 ml/kg/min. The data suggests that the mechanism for the WY 14,643 induced arginine lowering can almost entirely be attributed to a substantial reduction in *R_a_* (Fig. 4).

### Indirect calorimetry and assessment of urinary nitrogen elimination

**Figure 5:**
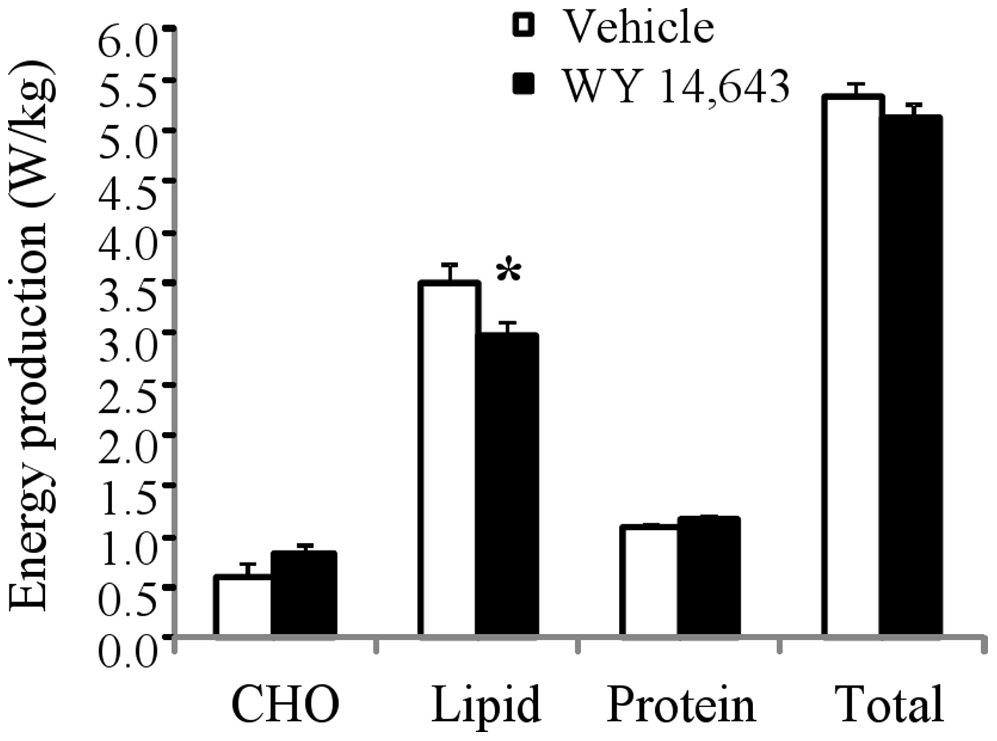
Estimated energy production from the oxidation of carbohydrate (CHO), lipid and protein, as well as the total energy expenditure (Total). Measurements were done in the fasting state following 4–5 week vehicle or WY 14,643 treatment in the rats. Values are given as means ± SEM.

WY 14,643 had no significant effect on neither *VO_2_*, *VCO_2_* nor *RQ* (Table 2). Despite the large changes in amino acid turnover described above, there was no apparent effect of WY 14,643 on nitrogen excretion (Table 2). Estimated energy production rates derived from the oxidation of carbohydrate (CHO), lipid and protein are summarized in Fig. 5. As expected in the fasting state, oxidation of lipid accounted for the majority (67%) of the energy production, however, proteins also accounted for a non-trivial fraction (∼21%). WY 14,643 had no apparent effect on protein oxidation, a consequence of the lack of effect on nitrogen excretion, nor did it affect net glucose oxidation (Fig. 5). Somewhat surprisingly, WY 14, 643 induced a minor reduction in lipid oxidation rate (P<0.05). WY 14,643 had no apparent affect on estimated energy expenditure (Total, Fig. 5) in the fasting state.

**Table 2:** Indirect calorimetry and urinary nitrogen excretion.

	Vehicle	WY 14,643
**Indirect calorimetry** a		
Body weight (g)	439±6	423±6
*VO_2_* (ml×kg^−1^×min^−1^)	16.0±0.2	16.6±0.4
*VCO_2_* (ml×kg^−1^×min^−1^)	12.2±0.2	12.6±0.3
*RQ*	0.760±0.006	0.761±0.006
**Nitrogen excretion rate** b(mmol×kg^−1^×day^−1^)	63.3±2.3	67.5±2.3

a Indirect calorimetry measurements were performed in 9 hr fasted animals following 4–5 weeks of treatment (n = 16/group).

b Based on measured urine volume and concentrations of urinary urea and creatinine in a subset of animals (n = 8/treatment group).

Values are given as means ± SEM.

### Decreased hepatic arginase activity

**Figure 6:**
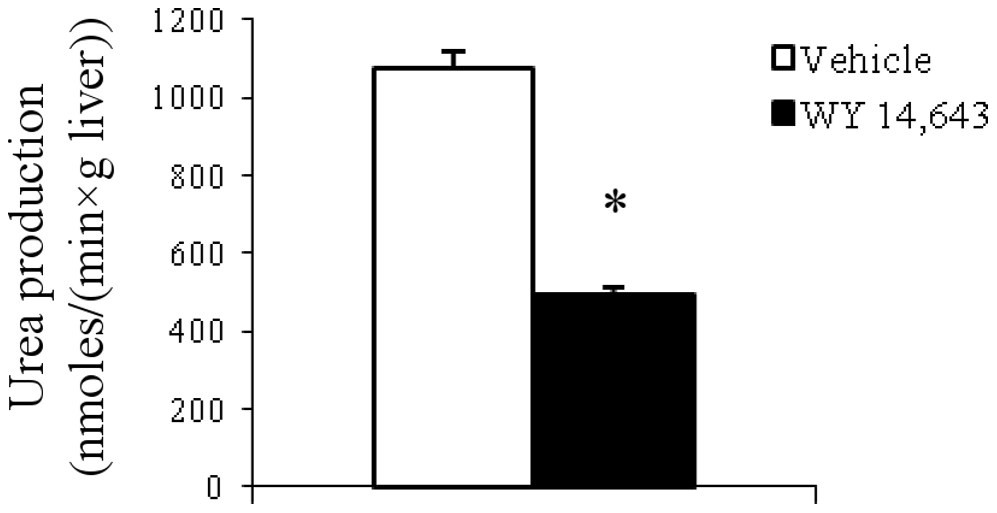
Hepatic arginase activity measurements in liver homogenates. Measurements in vehicle and WY 14,643 treated animals (n = 8/group). Values are given as means ± SEM. *P<0.05 versus vehicle.

Arginase activity was assessed based on the rate of production of the end product urea from a known amount of L-arginine added to the homogenate. The hepatic arginase activity was substantially lowered by WY 14,643 treatment (Fig. 6).

## Discussion

The current study extends our earlier work [2] by showing that the PPARα agonist WY 14,643 in rats: 1) markedly increased rates of appearance of serine and glycine, 2) reduced hepatic arginase activity, 3) suppressed the rate of appearance of arginine and 4) had no measurable effect on either protein catabolism or energy expenditure. We suggest that WY 14,643 has important effects on de novo synthesis of arginine, serine and glycine.

We gave the PPARα agonist WY 14,643 at a dose that induced desired metabolic effects without evidence of muscle wasting or reduction of food intake seen at higher doses [11], [12]. The well known pharmacodynamic effects of WY 14,643 [13], [14] were confirmed in the current study: significant reductions in plasma TG, FFA, evidence for an improvement in insulin sensitivity (Table 1) and liver enlargement with the magnitude of these changes comparing favourably to our previously published data [2].

In addition to these well established effects, WY 14,643 induced a widespread alteration in the plasma amino acid profile (Fig. 3). Fourteen of the 22 amino acids measured were significantly elevated by treatment with particularly large increases seen in serine, threonine and glycine and only 4 amino acids were lowered by WY 14,643, with arginine exhibiting the largest relative reduction. Overall the amino acid profile results were in agreement with our earlier study [2], which is noteworthy given that the previous data were obtained from conscious animals whereas the current data were obtained from anesthetized animals.

There are few reports in the literature with which to compare our amino acid data. Nonetheless there is evidence that some of the key changes observed are the result of specific modulation of PPARα function rather than non-specific effects of WY 14,643. In the study of Bjorndal and co-workers, tetradecylthioacetic acid (TTA) induced generally similar changes in the amino acid profile including increases in serine and glycine, as well as, arginine lowering and citrulline raising [15]. Further evidence is provided by studies of AVE8134 which suppressed plasma arginine and elevated citrulline [16], as well as the finding that PPARα deficiency in mice results in the opposite pattern [17].

Our indirect calorimetry measurements indicate that protein catabolism contributes significantly to energy expenditure (Fig. 5) in agreement with previous estimates of protein turnover energy costs [18], and therefore should not be ignored in attempts to quantitatively assess energy metabolism. Importantly, no treatment effects were observed on protein oxidation. *RQ* did not provide reliable information about lipid versus carbohydrate oxidation. Specifically, there was no difference between the *RQ* value in the WY 14,643 treated and vehicle groups, yet a small but significant, decrease in lipid oxidation was observed in WY 14,643 treated animals, perhaps, surprising considering the well established effects of PPARα agonists to up regulate the molecular machinery involved in lipid oxidation.

During regeneration of the liver in rat it has been found that plasma arginine is reduced [19], possibly a result of increased demand for polyamines which are required for the initiation of this process [20]. Also, in sepsis, arginine catabolism is increased and plasma levels drop mainly due to increased arginase activity [21]. In the case of WY 14,643 induced liver enlargement, a known effect of PPARα activation [22], hepatic arginase activity was actually down regulated.

**Figure 7:**
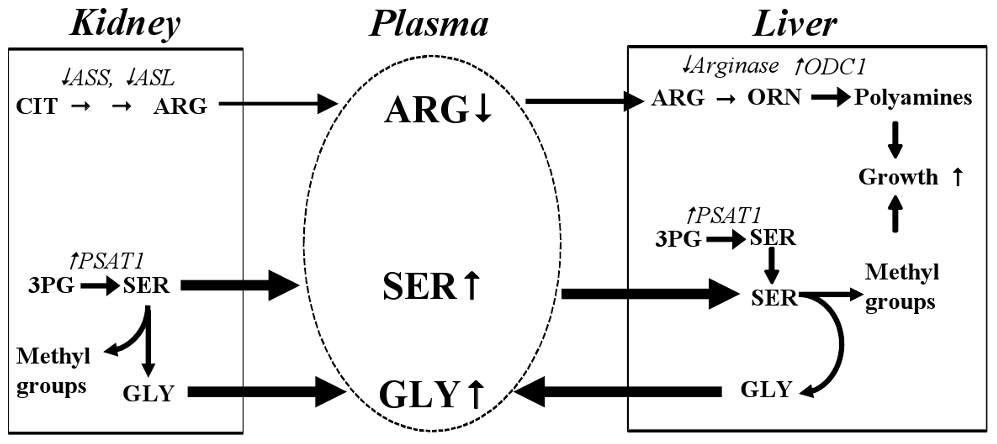
Schematic overview of suggested PPARα mediated changes in amino acid de novo synthesis. WY 14,643 reduced renal arginine (ARG) output, via down regulation of arginosuccinate synthetase (ASS) and argininosuccinate lyase (ASL), and increased tissue serine (SER) biosynthesis, via up regulation of phosphoserine aminotransferase 1 (PSAT1) with gene regulation previously shown in [2]. Available hepatic arginine is diverted to polyamine synthesis via altered gene expression including ornithine decarboxylase 1 (ODC1) up regulation [2]. Serine is used to provide methyl group transfer to support expected PPARα-mediated liver growth and in the process generating glycine (GLY). CIT, citrulline; ORN, ornithine; 3PG, 3-phosphoglycerate.

The current data shows that reduced levels of plasma arginine could largely be accounted for by reduced *R_a_*, but what could be the mechanism? The renal pathway forming arginine from circulating citrulline is the primary pathway responsible for maintenance of plasma arginine levels [23]–[25]. Citrulline levels in plasma were not a limiting factor in our experiment and thus not likely contributing to arginine lowering. A potential mechanism for WY 14,643 induced suppression of arginine *R_a_* could be down regulation of renal arginosuccinate synthetase (ASS) and argininosuccinate lyase (ASL), two enzymes involved in the urea cycle converting citrulline to arginine (Fig. 7). We have previously observed downregulation in these genes in liver in response to WY 14,643 [2]. We have not explicitly analyzed gene regulation in kidney, however, kidney specific effects of PPARα are known from the literature [26].

The WY14643 induced increases in serine and glycine *R_a_* may be the result of increased de novo synthesis of serine via the phosphorylated intermediate pathway which generates glycine as a byproduct (Fig. 7). This is suggested by similarities between current data and a study by Kalhan et al. [27] who showed that protein restriction 1) increased plasma glycine and serine 2) due to increases in *R_a_* and 3) that the increase in serine de novo synthesis could quantitatively account for the increase in serine *R_a_*. The link to PPARα was made by their observation that hepatic PPARα, was up regulated. Furthermore they found genes involved in serine de novo synthesis including phosphoserine aminotransferase 1 (PSAT1) were markedly up regulated in liver and kidney while genes involved in the urea cycle were down regulated [27]. Similarly in our previous study [2] we demonstrated increased expression of hepatic PSAT1 and down regulation of genes of the urea cycle.

In conclusion, this study confirms the observations made in our initial study showing that the selective PPARα agonist WY 14,643 has profound effects on amino acid metabolism. Here we extend our earlier observations by demonstrating changes in a specific subset of amino acids (serine, glycine and arginine). We suggest that these changes in appearance rate reflect PPARα mediated changes in de novo synthesis.

We declare a competing interest as all authors are currently employed by or have been supported financially by AstraZeneca, the sponsor of this study.
